# Supplementary figures and images for: Publication language and the estimate of treatment effects of physical therapy on balance and postural control after stroke in meta-analyses of randomised controlled trials
Source: PLoS One. 2020 Mar 9;15(3):e0229822. doi: 10.1371/journal.pone.0229822 (PMC7062257; doi:10.1371/journal.pone.0229822)

**S1 Fig. Histogram of studies according to the language of publication for studies included**


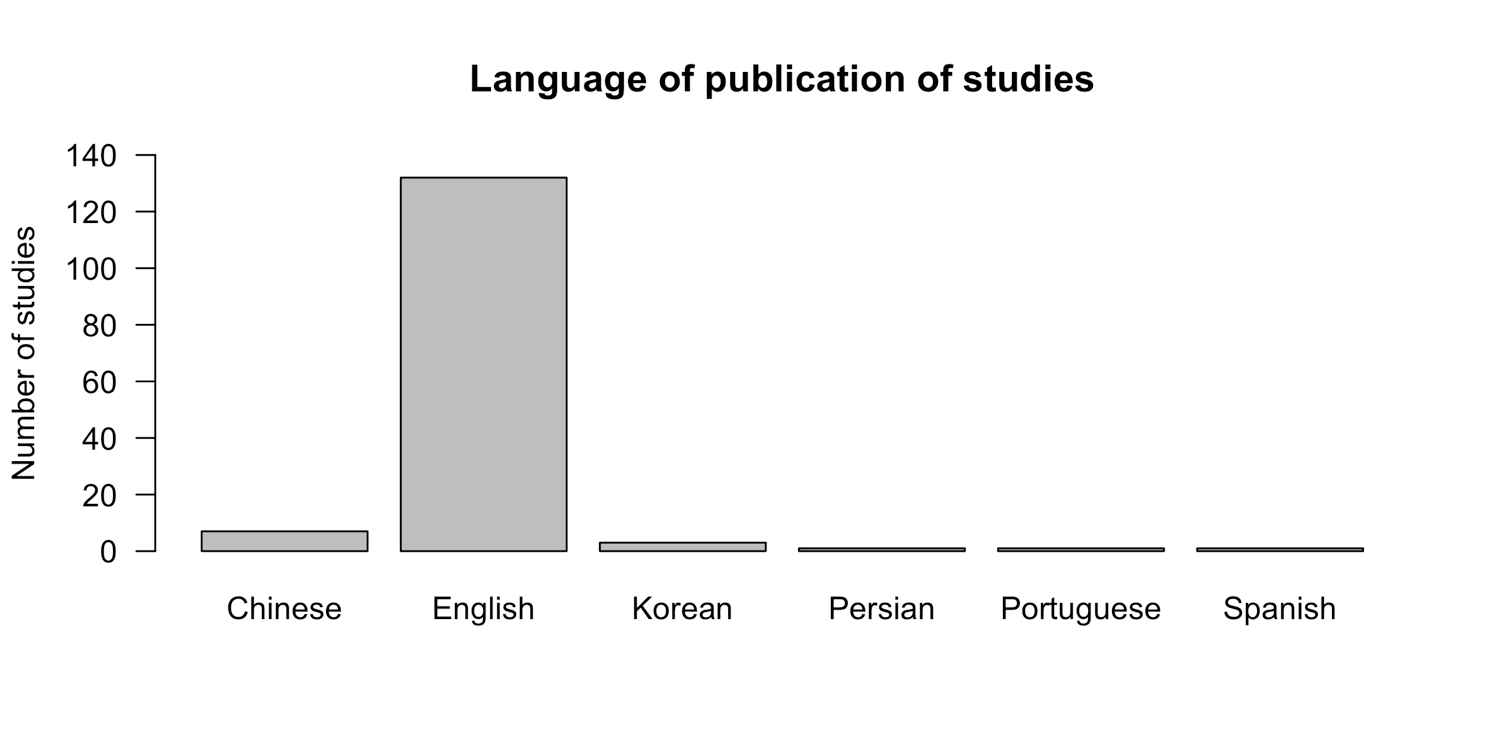

Supplement: S1 Fig — (DOCX) [file pone.0229822.s002.docx]
