# Supplementary material for: Publication language and the estimate of treatment effects of physical therapy on balance and postural control after stroke in meta-analyses of randomised controlled trials
Source: PLoS One. 2020 Mar 9;15(3):e0229822. doi: 10.1371/journal.pone.0229822 (PMC7062257; doi:10.1371/journal.pone.0229822)
Supplement: S3 Fig — (DOCX) [file pone.0229822.s004.docx]

**S3 Fig. Risk of bias summary: review authors' judgements about each risk of bias item for each included study**.

Judgements about risk of bias: Green colour means low risk, yellow colour means unclear risk and red colour means high risk.
