# Supplementary material for: Publication language and the estimate of treatment effects of physical therapy on balance and postural control after stroke in meta-analyses of randomised controlled trials
Source: PLoS One. 2020 Mar 9;15(3):e0229822. doi: 10.1371/journal.pone.0229822 (PMC7062257; doi:10.1371/journal.pone.0229822)
Supplement: S4 Fig — (DOCX) [file pone.0229822.s005.docx]

**S4 Fig. Summary of overall score of risk of bias**


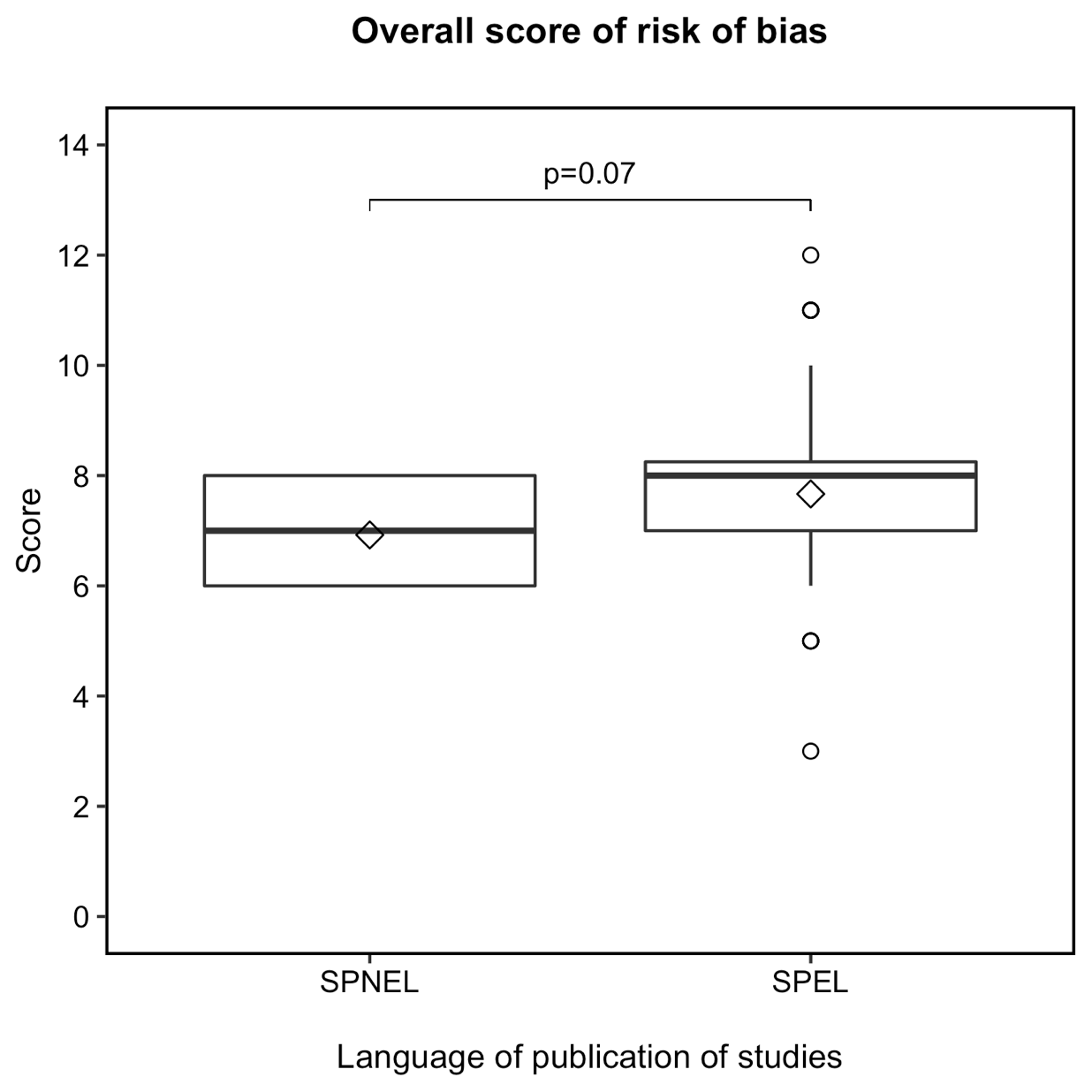


SPEL, studies published in English language; SPNEL, studies published in non-English language
