# Supplementary material for: Publication language and the estimate of treatment effects of physical therapy on balance and postural control after stroke in meta-analyses of randomised controlled trials
Source: PLoS One. 2020 Mar 9;15(3):e0229822. doi: 10.1371/journal.pone.0229822 (PMC7062257; doi:10.1371/journal.pone.0229822)
Supplement: S5 Fig — (DOCX) [file pone.0229822.s006.docx]

**S5 Fig. Funnel plot for all studies (SPEL and SPNEL)**

**S5A Fig. Funnel plot of comparison PT versus no treatment for all studies (SPEL and SPNEL)**


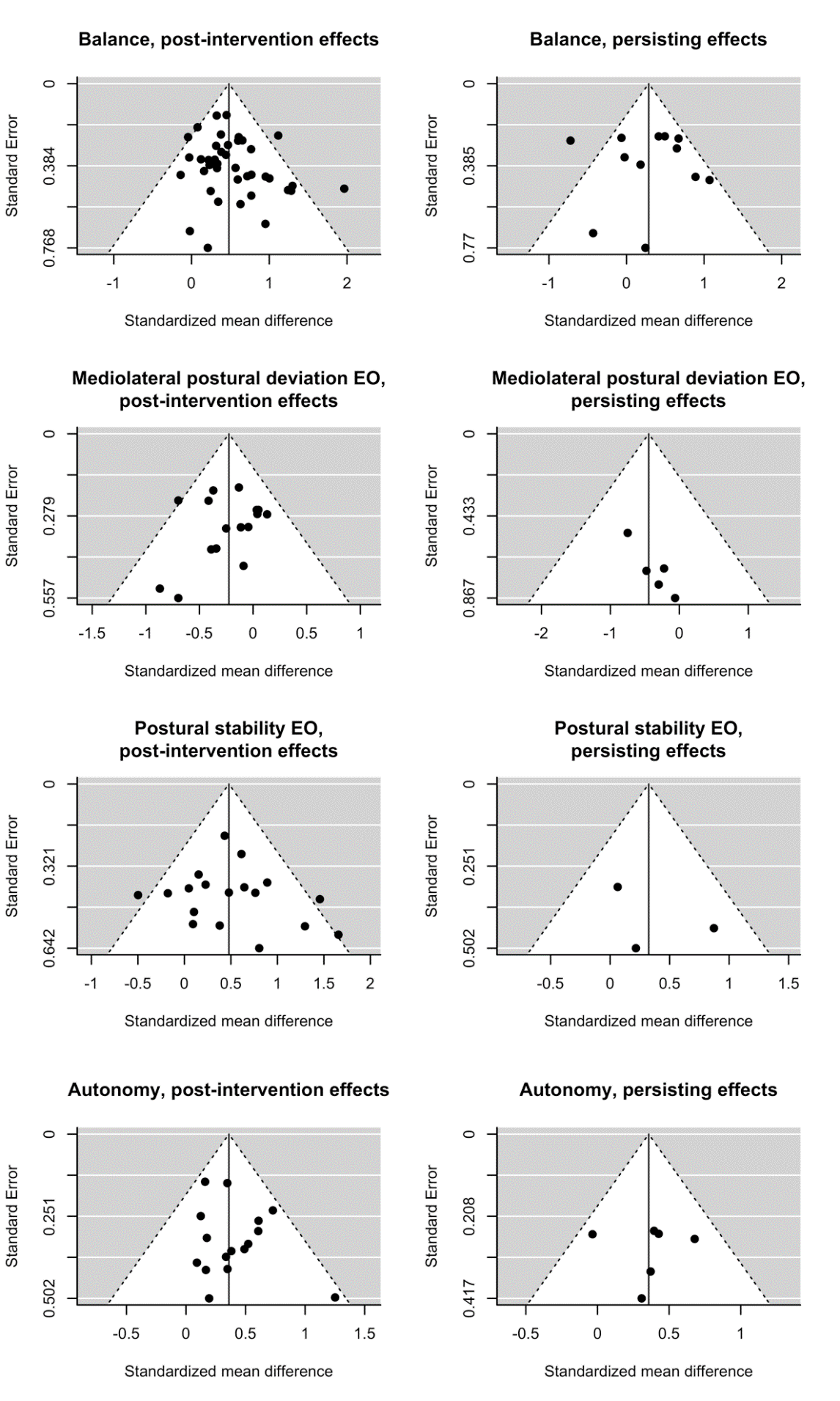


Dotted line: 95% confidence interval; black filled circle: study

EO, eyes open; SPEL, studies published in English language; SPNEL, studies published in non-English language

**S5B Fig. Funnel plot of comparison PT versus sham treatment or usual care for all studies (SPEL and SPNEL)**


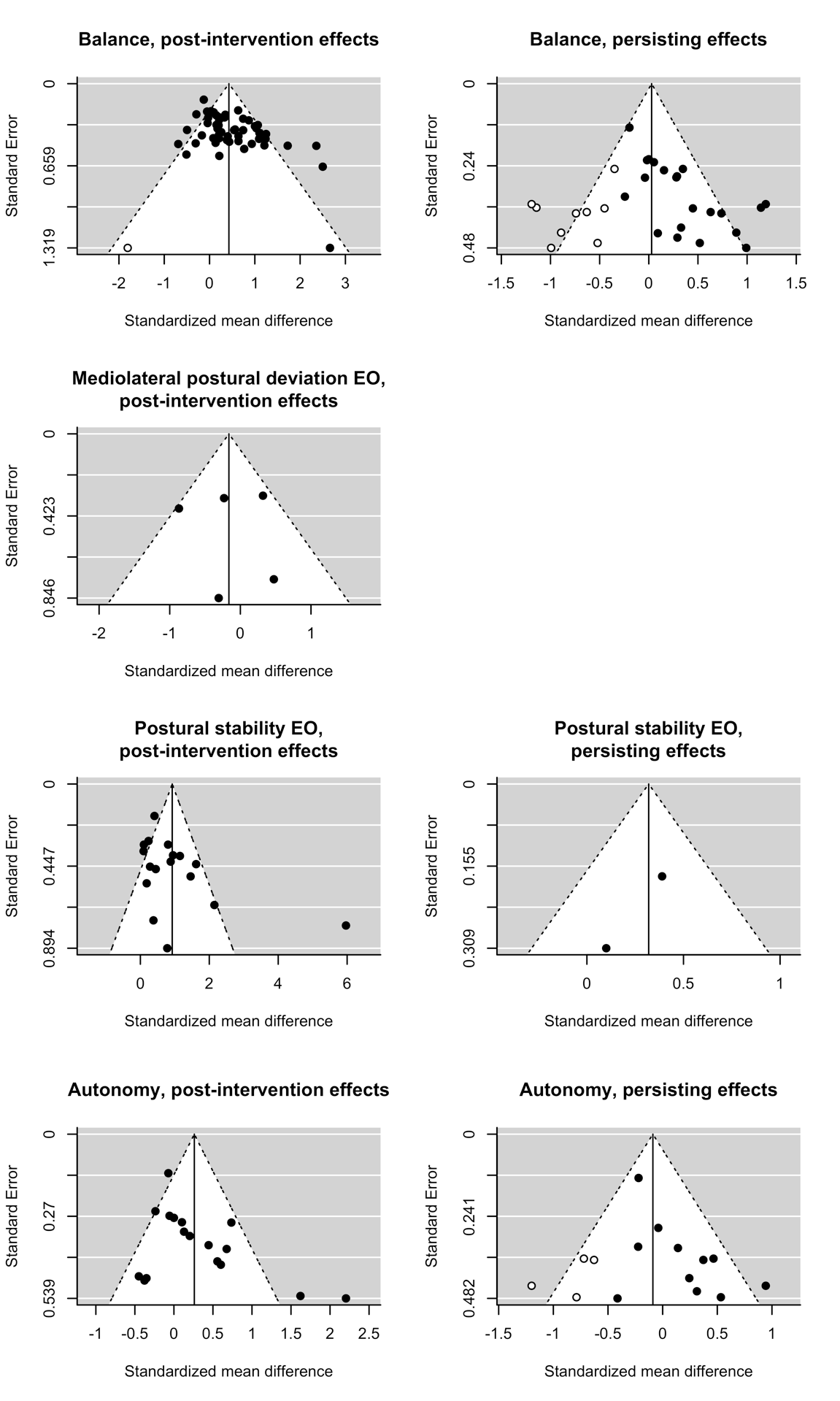


Dotted line: 95% confidence interval; black filled circle: study; white filled circle: “missing” study

EO, eyes open; SPEL, studies published in English language; SPNEL, studies published in non-English language
