# Supplementary material for: Publication language and the estimate of treatment effects of physical therapy on balance and postural control after stroke in meta-analyses of randomised controlled trials
Source: PLoS One. 2020 Mar 9;15(3):e0229822. doi: 10.1371/journal.pone.0229822 (PMC7062257; doi:10.1371/journal.pone.0229822)
Supplement: S6 Fig — (DOCX) [file pone.0229822.s007.docx]

**S6 Fig. Funnel plot for all studies for SPEL only**

**S6A Fig. Funnel plot of comparison PT versus no treatment for SPEL only**


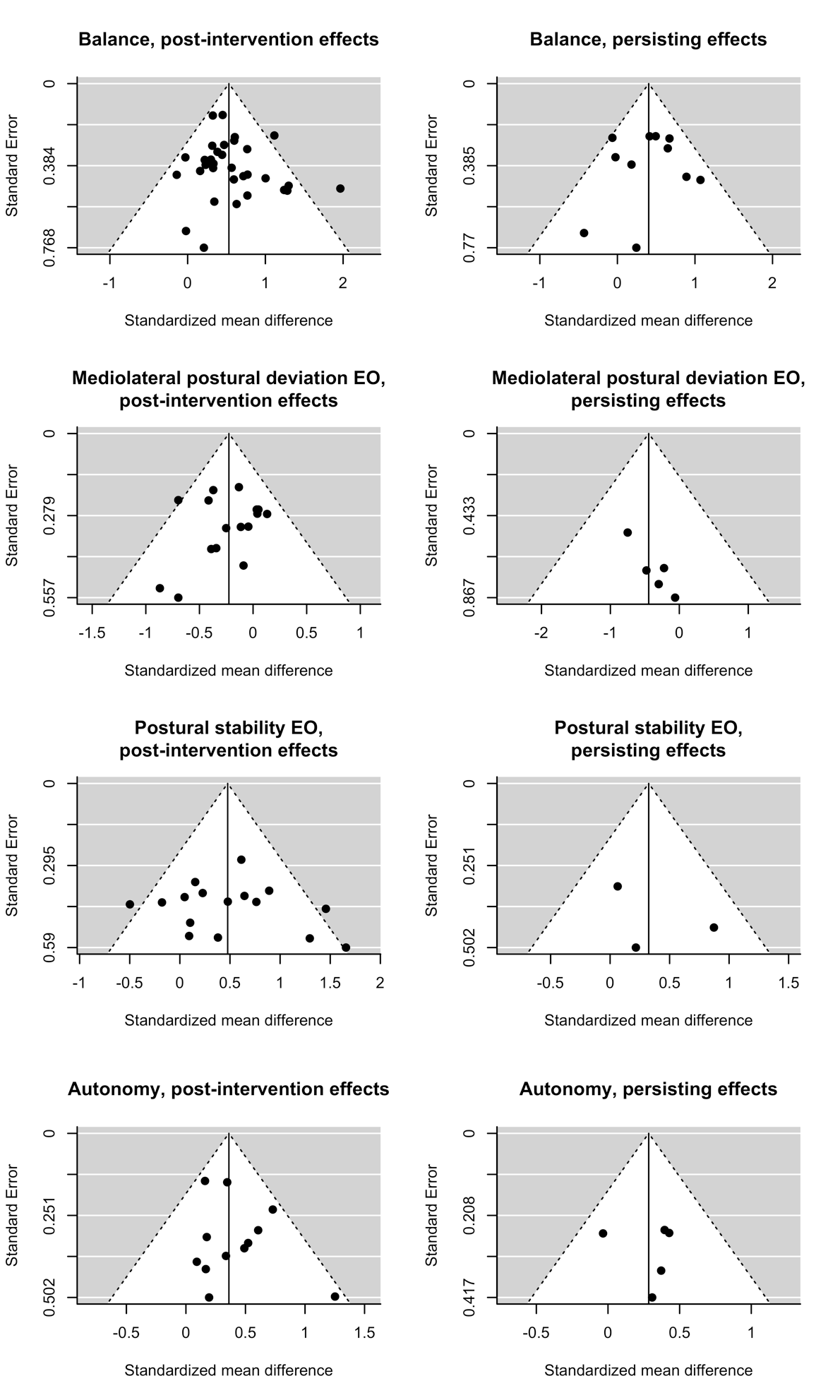


Dotted line: 95% confidence interval; black filled circle: study

EO, eyes open; SPEL, studies published in English language

**S6B Fig. Funnel plot of comparison PT versus sham treatment or usual care** **for SPEL only**


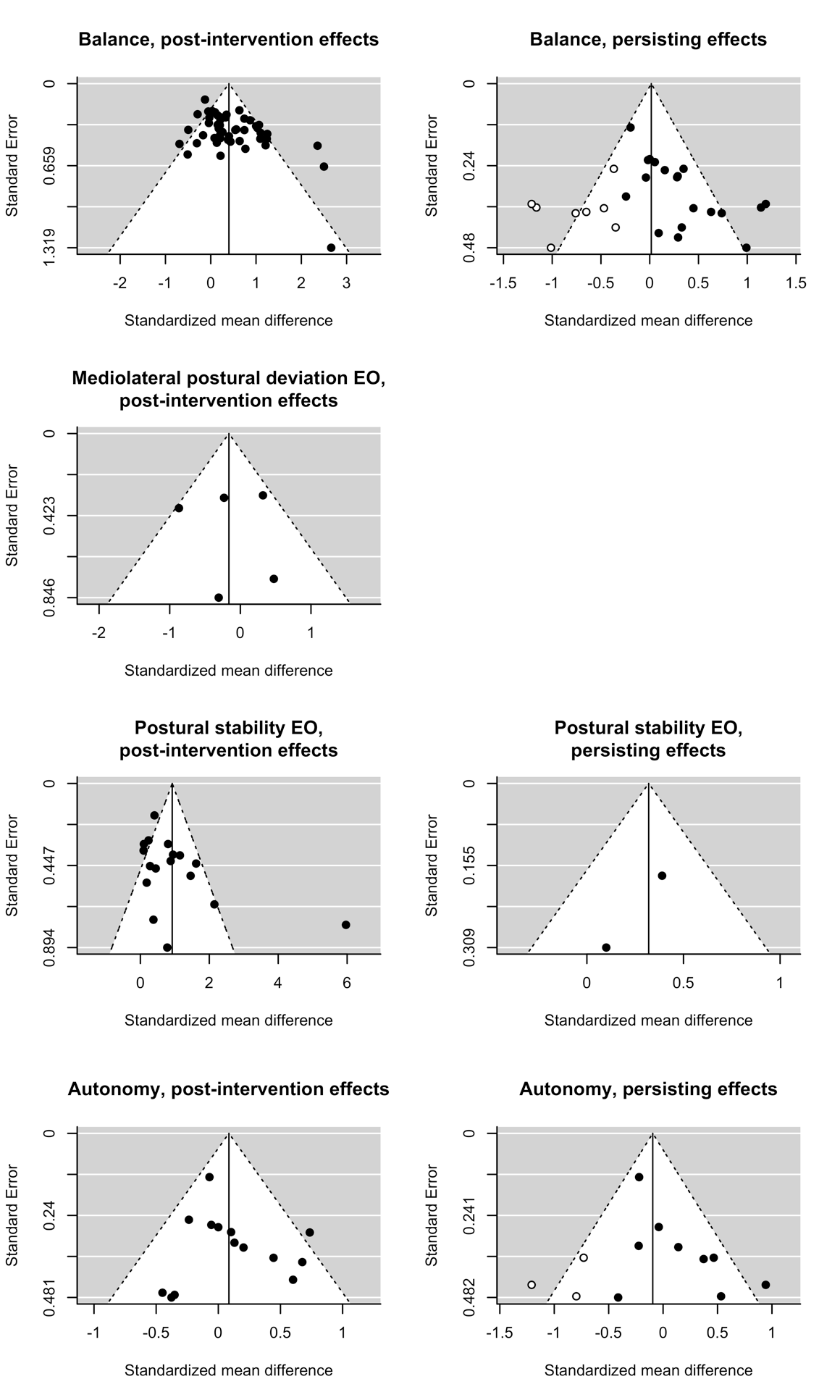


Dotted line: 95% confidence interval; black filled circle: study; white filled circle: “missing” study

EO, eyes open; SPEL, studies published in English language; ST, sham treatment; UC, usual care
