# Supplementary material for: Publication language and the estimate of treatment effects of physical therapy on balance and postural control after stroke in meta-analyses of randomised controlled trials
Source: PLoS One. 2020 Mar 9;15(3):e0229822. doi: 10.1371/journal.pone.0229822 (PMC7062257; doi:10.1371/journal.pone.0229822)

**S9 Fig. Forest plots of physical therapy versus sham treatment or usual care. Subgroup: Language of publication of studies**

**S9A Fig. Forest plot of physical therapy versus sham treatment or usual care. Outcome: Balance, immediate effects. Subgroup: Language of publication of studies**


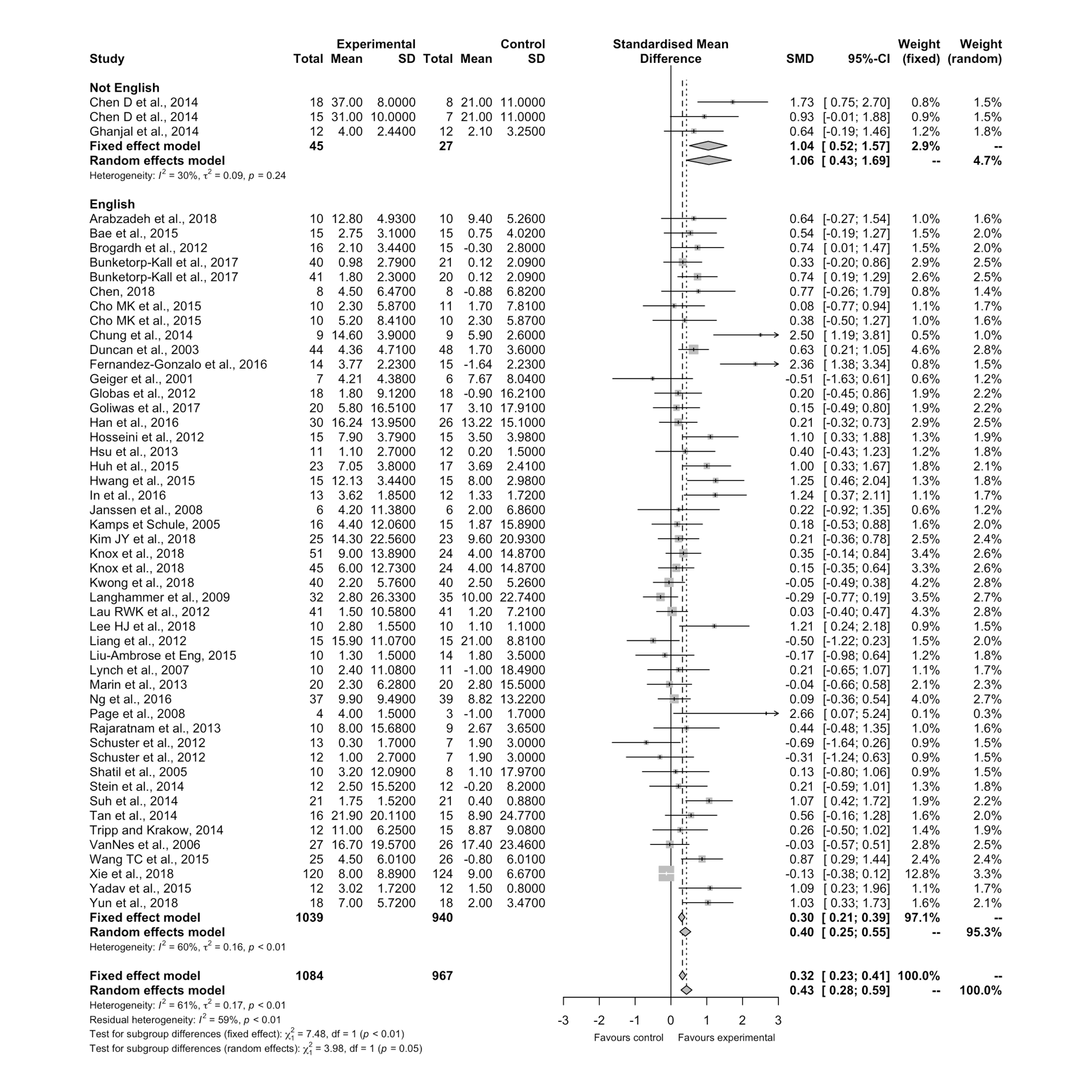


**S9B Fig. Forest plot of physical therapy versus sham treatment or usual care. Outcome: Balance, persisting effects. Subgroup: Language of publication of studies**


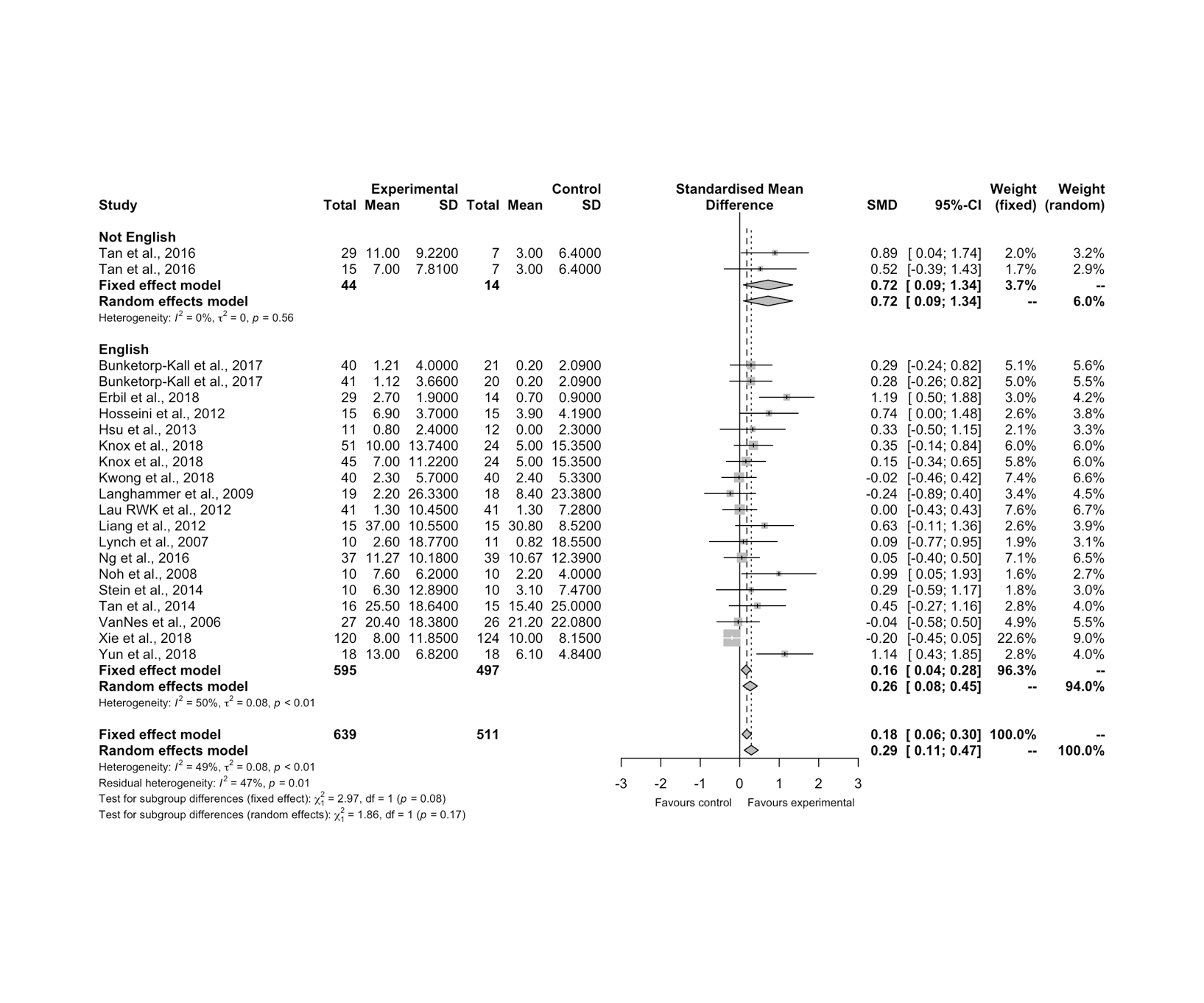


**S9C Fig. Forest plot of physical therapy versus sham treatment or usual care. Outcome: Mediolateral postural deviation EO, immediate effects. Subgroup: Language of publication of studies**


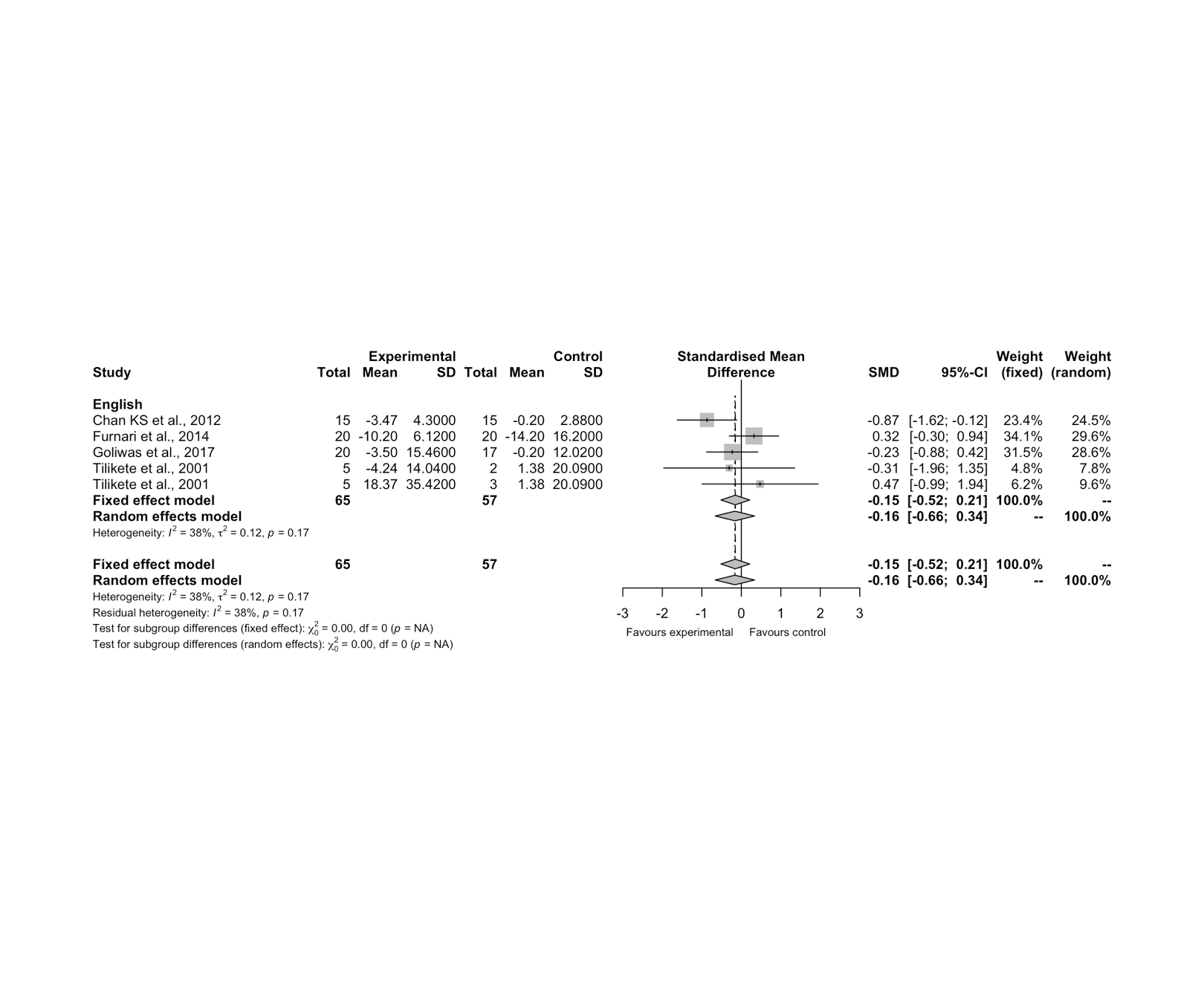


**S9D Fig. Forest plot of physical therapy versus sham treatment or usual care. Outcome: Postural stability EO, immediate effects. Subgroup: Language of publication of studies**


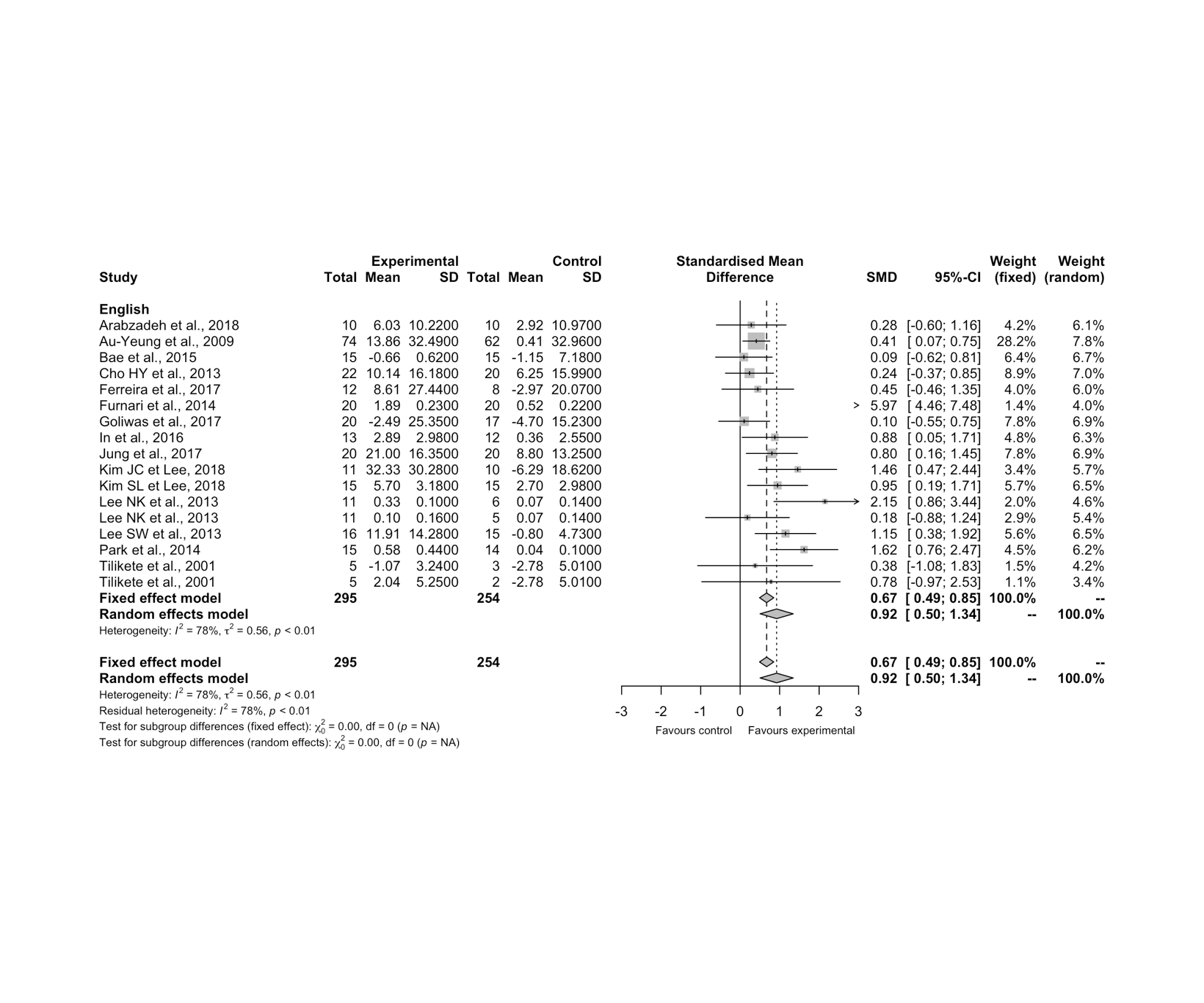


**S9E Fig. Forest plot of physical therapy versus sham treatment or usual care. Outcome: Postural stability EO, persisting effects. Subgroup: Language of publication of studies**


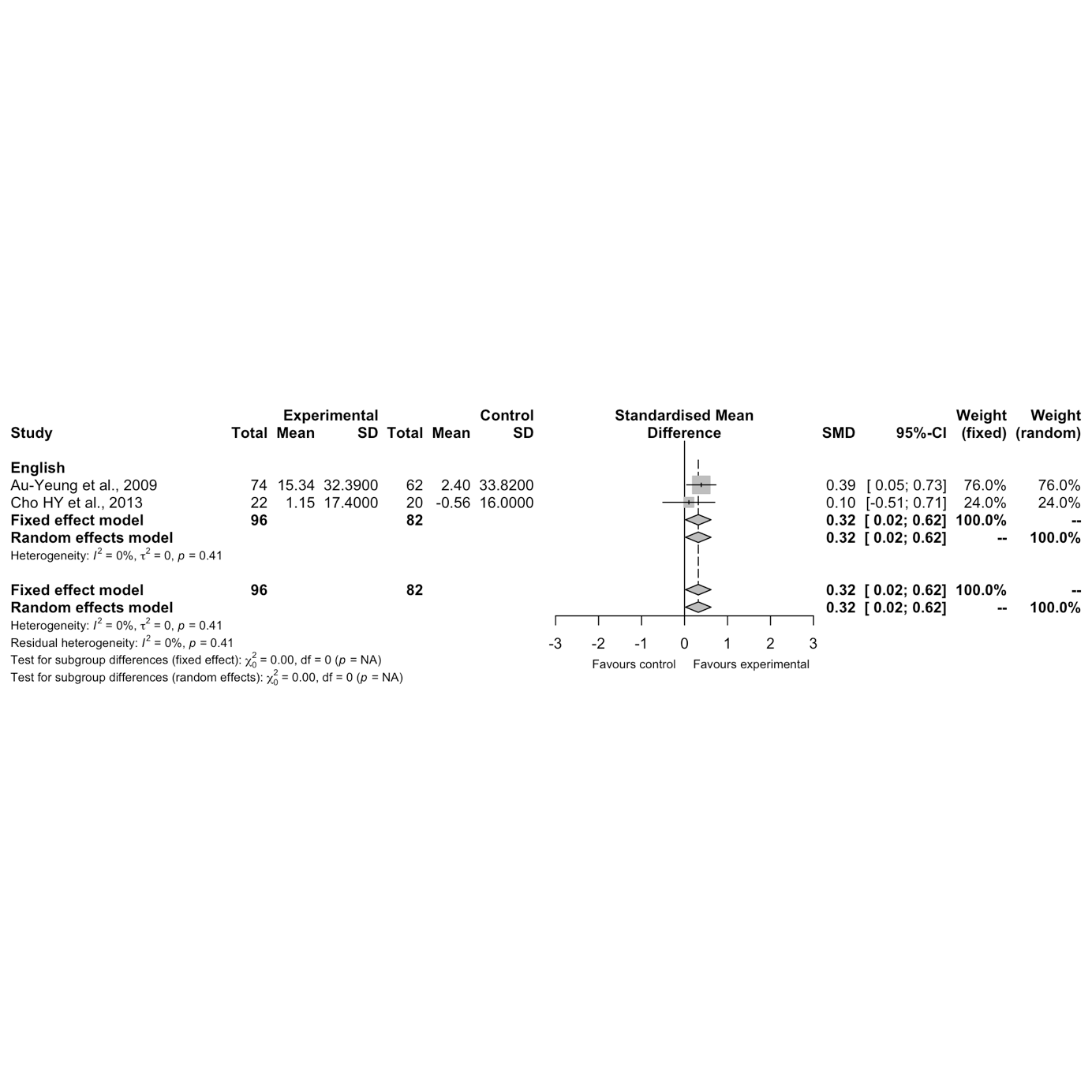


**S9F Fig. Forest plot of physical therapy versus sham treatment or usual care. Outcome: Autonomy, immediate effects. Subgroup: Language of publication of studies**


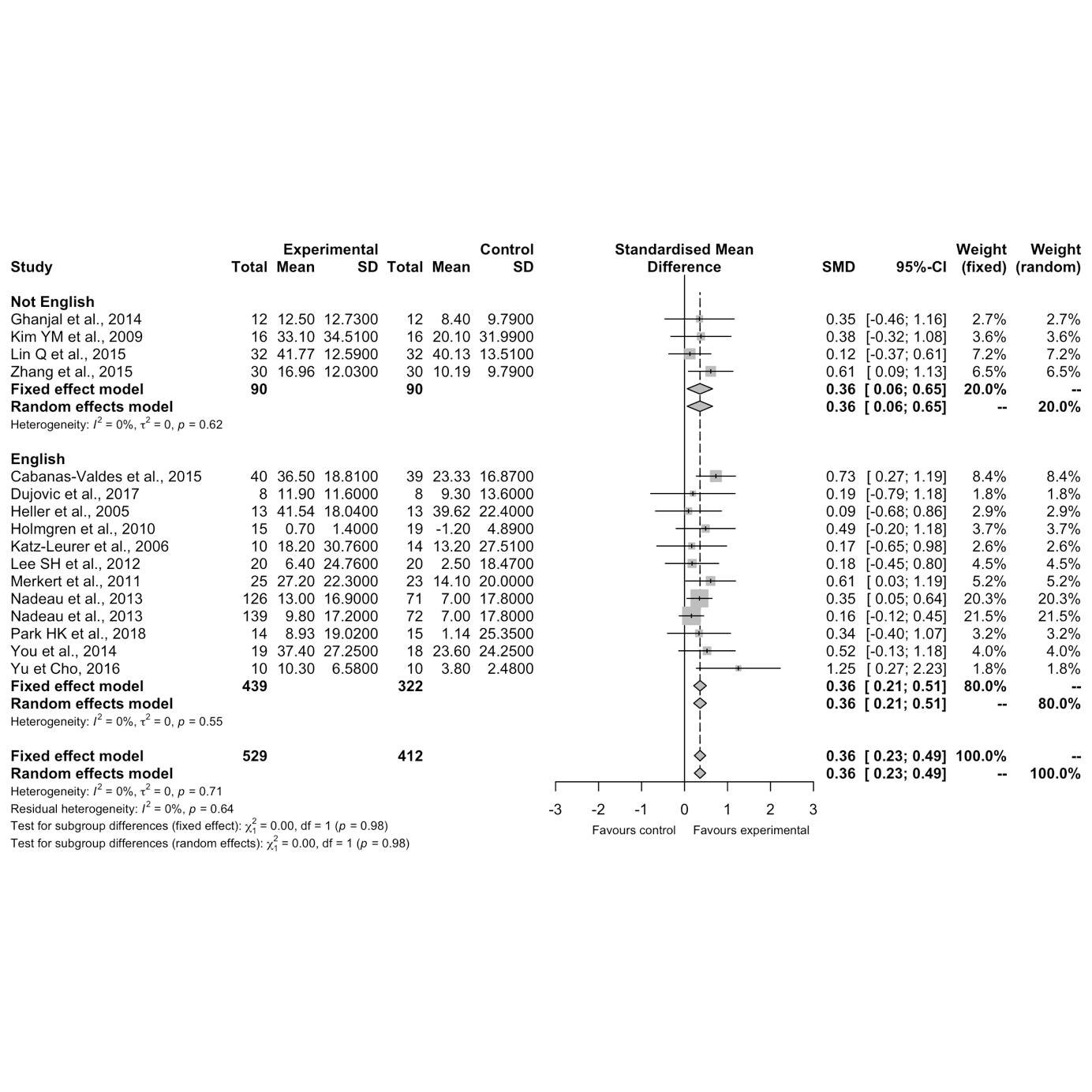


**S9G Fig. Forest plot of physical therapy versus sham treatment or usual care. Outcome: Autonomy, persisting effects. Subgroup: Language of publication of studies**


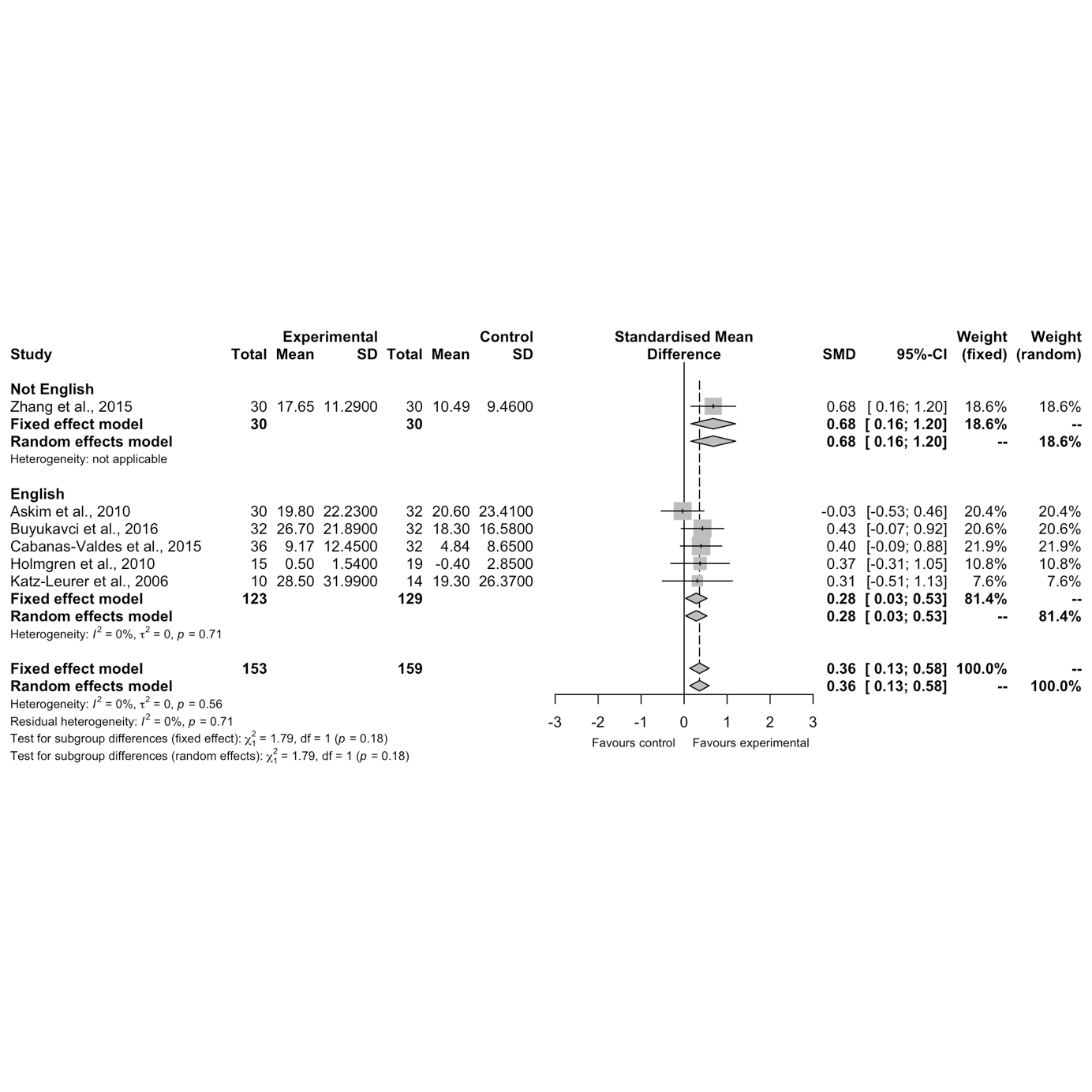

Supplement: S9 Fig — Subgroup: Language of publication of studies. (DOCX) [file pone.0229822.s010.docx]
