# Supplementary material for: Publication language and the estimate of treatment effects of physical therapy on balance and postural control after stroke in meta-analyses of randomised controlled trials
Source: PLoS One. 2020 Mar 9;15(3):e0229822. doi: 10.1371/journal.pone.0229822 (PMC7062257; doi:10.1371/journal.pone.0229822)
Supplement: S2 Table — (DOCX) [file pone.0229822.s012.docx]

**S2 Table. Summary of risk of bias of studies included**

| SPEL only | | | |
| --- | --- | --- | --- |
| Risk of bias | Number of studies with high risk | Number of studies with unclear risk | Number of studies with low risk |
| Random sequence generation, n (%) | 0 (0%) | 58 (44%) | 74 (56%) |
| Allocation concealment, n (%) | 18 (14%) | 95 (72%) | 19 (14%) |
| Blinding of outcome assessment, n (%) | 13 (10%) | 55 (42%) | 64 (48%) |
| Incomplete outcome data, n (%) | 71 (54%) | 36 (27%) | 25 (19%) |
| Blinding of patients and therapists, n (%) | 116 (88%) | 14 (11%) | 2 (1%) |
| Selective reporting, n (%) | 20 (15%) | 89 (68%) | 23 (17%) |
| Other bias, n (%) | 2 (2%) | 8 (6%) | 122 (92%) |

| SPNEL only | | | |
| --- | --- | --- | --- |
| Risk of bias | Number of studies with high risk | Number of studies with unclear risk | Number of studies with low risk |
| Random sequence generation, n (%) | 0 (0%) | 7 (54%) | 6 (46%) |
| Allocation concealment, n (%) | 0 (0%) | 13 (100%) | 0 (0%) |
| Blinding of outcome assessment, n (%) | 0 (0%) | 13 (100%) | 0 (0%) |
| Incomplete outcome data, n (%) | 3 (23%) | 10 (77%) | 0 (0%) |
| Blinding of patients and therapists, n (%) | 13 (100%) | 0 (0%) | 0 (0%) |
| Selective reporting, n (%) | 2 (15%) | 11 (85%) | 0 (0%) |
| Other bias, n (%) | 0 (0%) | 2 (15%) | 11 (85%) |

| Difference between SPEL and SPNEL: p-value of Chi^2^ test | | |
| --- | --- | --- |
| Item of risk of bias | Low risk versus high and unclear risks | Proportion of unclear |
| Random sequence generation, n (%) | p=0.69 | p=0.69 |
| Allocation concealment, n (%) | p=0.30 | p=0.06 |
| Blinding of outcome assessment, n (%) | p=0.002* | p<0.001* |
| Incomplete outcome data, n (%) | p=0.18 | p<0.001* |
| Blinding of patients and therapists, n (%) | p=1.00 | p=0.46 |
| Selective reporting, n (%) | p=0.21 | p=0.34 |
| Other bias, n (%) | p=0.65 | p=0.49 |

^*^ Significant difference (p≤0.05) between SPEL and SPNEL

SPEL, studies published in English language; SPNEL, studies published in non-English language
