# Supplementary material for: Publication language and the estimate of treatment effects of physical therapy on balance and postural control after stroke in meta-analyses of randomised controlled trials
Source: PLoS One. 2020 Mar 9;15(3):e0229822. doi: 10.1371/journal.pone.0229822 (PMC7062257; doi:10.1371/journal.pone.0229822)
Supplement: S3 Table — (DOCX) [file pone.0229822.s013.docx]

**S3 Table. Summary of overall score of risk of bias**

| Overall score of risk of bias | Mean (SD) | Median | Min-max | Wilcoxon rank sum test |
| --- | --- | --- | --- | --- |
| SPEL | 7.67 (1.53) | 8 | 3-12 | 0.07 |
| SPNEL | 6.92 (0.86) | 7 | 6-8 |  |

SPEL, studies published in English language; SPNEL, studies published in non-English language
