# Supplementary material for: Publication language and the estimate of treatment effects of physical therapy on balance and postural control after stroke in meta-analyses of randomised controlled trials
Source: PLoS One. 2020 Mar 9;15(3):e0229822. doi: 10.1371/journal.pone.0229822 (PMC7062257; doi:10.1371/journal.pone.0229822)
Supplement: S4 Table — (DOCX) [file pone.0229822.s014.docx]

**S4 Table. Results of Egger tests detecting bias of publication for all studies (SPEL and SPNEL)**

| Comparison | Outcome | Post-intervention or persisting effects | Number of points | P value |
| --- | --- | --- | --- | --- |
| PT vs NT | Balance | Post-intervention effects | 40 | 0.10 |
| PT vs NT | Mediolateral postural deviation EO, crossover RCTs | Post-intervention effects | 16 | 0.70 |
| PT vs NT | Postural stability EO | Post-intervention effects | 18 | 0.44 |
| PT vs NT | Autonomy | Post-intervention effects | 16 | 0.33 |
| PT vs NT | Balance | Persisting effects | 12 | 0.93 |
| PT vs NT | Mediolateral postural deviation EO | Persisting effects | 5 | 0.45 |
| PT vs NT | Postural stability EO | Persisting effects | 3 | 0.58 |
| PT vs NT | Autonomy | Persisting effects | 6 | 0.98 |
| PT vs ST/UC | Balance | Post-intervention effects | 51 | <0.01 |
| PT vs ST/UC | Mediolateral postural deviation EO, parallel RCTs | Post-intervention effects | 5 | 0.83 |
| PT vs ST/UC | Postural stability EO | Post-intervention effects | 17 | <0.01 |
| PT vs ST/UC | Autonomy | Post-intervention effects | 17 | 0.03 |
| PT vs ST/UC | Balance | Persisting effects | 21 | <0.01 |
| PT vs ST/UC | Mediolateral postural deviation EO | Persisting effects | 0 | NC |
| PT vs ST/UC | Postural stability EO | Persisting effects | 2 | NC |
| PT vs ST/UC | Autonomy | Persisting effects | 11 | <0.01 |

EO, eyes open; NC, not calculable; RCT, randomized controlled trial; SPEL, studies published in English language; SPNEL, studies published in non-English language; ST, sham treatment; UC, usual care; vs, versus
