# Supplementary material for: Publication language and the estimate of treatment effects of physical therapy on balance and postural control after stroke in meta-analyses of randomised controlled trials
Source: PLoS One. 2020 Mar 9;15(3):e0229822. doi: 10.1371/journal.pone.0229822 (PMC7062257; doi:10.1371/journal.pone.0229822)
Supplement: S5 Table — (DOCX) [file pone.0229822.s015.docx]

**S5 Table. Results of Egger test detecting bias of publication for SPEL only**

| Comparison | Outcome | Post-intervention or persisting effects | Number of points | P value |
| --- | --- | --- | --- | --- |
| PT vs NT | Balance | Post-intervention effects | 32 | 0.31 |
| PT vs NT | Mediolateral postural deviation EO, crossover RCTs | Post-intervention effects | 16 | 0.70 |
| PT vs NT | Postural stability EO | Post-intervention effects | 16 | 0.43 |
| PT vs NT | Autonomy | Post-intervention effects | 12 | 0.32 |
| PT vs NT | Balance | Persisting effects | 11 | 0.83 |
| PT vs NT | Mediolateral postural deviation EO | Persisting effects | 5 | 0.45 |
| PT vs NT | Postural stability EO | Persisting effects | 3 | 0.57 |
| PT vs NT | Autonomy | Persisting effects | 5 | 0.87 |
| PT vs ST/UC | Balance | Post-intervention effects | 48 | <0.01 |
| PT vs ST/UC | Mediolateral postural deviation EO, parallel RCTs | Post-intervention effects | 5 | 0.83 |
| PT vs ST/UC | Postural stability EO | Post-intervention effects | 17 | <0.01 |
| PT vs ST/UC | Autonomy | Post-intervention effects | 14 | 0.62 |
| PT vs ST/UC | Balance | Persisting effects | 19 | <0.001 |
| PT vs ST/UC | Mediolateral postural deviation EO | Persisting effects | 0 | NC |
| PT vs ST/UC | Postural stability EO | Persisting effects | 2 | NC |
| PT vs ST/UC | Autonomy | Persisting effects | 9 | 0.02 |

EO, eyes open; NC, not calculable; RCT, randomized controlled trial; SPEL, studies published in English language; ST, sham treatment; UC, usual care; vs, versus
