# Supplementary material for: Publication language and the estimate of treatment effects of physical therapy on balance and postural control after stroke in meta-analyses of randomised controlled trials
Source: PLoS One. 2020 Mar 9;15(3):e0229822. doi: 10.1371/journal.pone.0229822 (PMC7062257; doi:10.1371/journal.pone.0229822)
Supplement: S6 Table — (DOCX) [file pone.0229822.s016.docx]

**S6 Table. Summary of comparisons of intervention**

| Characteristics | SPEL | SPNEL | Chi^2^ test |
| --- | --- | --- | --- |
| Design of comparison |  |  | p=0.03 |
| Direct comparison (e.g. A vs B), n (%) | 65 (42%) | 2 (12%) |  |
| Comparison “on-top” or “add-on” (e.g. A+B versus C+B), n (%) | 90 (58%) | 15 (88%) |  |
| Comparator group (control treatment) |  |  | p=0.85 |
| No treatment, n (%) | 82 (53%) | 9 (53%) |  |
| Sham treatment, n (%) | 29 (19%) | 4 (23.5%) |  |
| Usual care, n (%) | 44 (28%) | 4 (23.5%) |  |

SPEL, studies published in English language; SPNEL, studies published in non-English language; vs, versus
