# Supplementary material for: Publication language and the estimate of treatment effects of physical therapy on balance and postural control after stroke in meta-analyses of randomised controlled trials
Source: PLoS One. 2020 Mar 9;15(3):e0229822. doi: 10.1371/journal.pone.0229822 (PMC7062257; doi:10.1371/journal.pone.0229822)
Supplement: S7 Table — (DOCX) [file pone.0229822.s017.docx]

**S7 Table. Summary of categories of physical therapy investigated in studies included**

| Categories of PT | SPEL | |  | SPNEL | |  |
| --- | --- | --- | --- | --- | --- | --- |
|  | Number of comparisons | Compared to all categories in SPEL | Compared to SPEL and SPNEL of the category | Number of comparisons | Compared to all categories in SPNEL | Compared to SPEL and SPNEL of the category |
| Acupuncture | 1 | 1% | 20% | 4 | 24% | 80% |
| Assistive devices | 21 | 14% | 100% | 0 | 0% | 0% |
| Constraint-induced therapy | 3 | 2% | 75% | 1 | 6% | 25% |
| Functional task-training | 51 | 33% | 91% | 5 | 29% | 9% |
| Functional task-training + other intervention | 1 | 1% | 100% | 0 | 0% | 0% |
| Functional task-training and musculoskeletal intervention and/or cardiopulmonary intervention | 29 | 19% | 100% | 0 | 0% | 0% |
| Functional task-training and neurophysiological intervention | 2 | 1% | 100% | 0 | 0% | 0% |
| Musculoskeletal intervention and body awareness therapy | 1 | 1% | 100% | 0 | 0% | 0% |
| Musculoskeletal intervention: active strengthening | 8 | 5% | 89% | 1 | 6% | 11% |
| Musculoskeletal intervention: electrostimulation | 11 | 7% | 69% | 5 | 29% | 31% |
| Musculoskeletal intervention: immobilization | 4 | 3% | 100% | 0 | 0% | 0% |
| Musculoskeletal intervention: mobilisation | 2 | 1% | 100% | 0 | 0% | 0% |
| Respiratory training | 2 | 1% | 100% | 0 | 0% | 0% |
| Sensory intervention | 19 | 12% | 100% | 0 | 0% | 0% |
| Visual and occulomotor training | 0 | 0% | 0% | 1 | 6% | 100% |

PT, physical therapy; SPEL, studies published in English language; SPNEL, studies published in non-English language
