# Supplementary material for: Publication language and the estimate of treatment effects of physical therapy on balance and postural control after stroke in meta-analyses of randomised controlled trials
Source: PLoS One. 2020 Mar 9;15(3):e0229822. doi: 10.1371/journal.pone.0229822 (PMC7062257; doi:10.1371/journal.pone.0229822)
Supplement: S8 Table — (DOCX) [file pone.0229822.s018.docx]

**S8 Table. Summary of duration of physical therapy compared**

|  | SPEL | SPNEL | Difference |
| --- | --- | --- | --- |
| Mean duration of session in minutes | 33.0 ± 70.2 / 20.0 / 0.5-600.0 | 34.3 ± 29.8 / 30.0 / 15.0-100.0 | 0.35^a^ |
| Number of sessions by week | 3.1 ± 1.9 / 3.0 / 1.0-7.5 | 4.6 ± 1.6 / 5.0 / 1.7-7.0 | 0.04^a*^ |
| Number of weeks | 3.6 ± 3.0 / 3.0 / 1.0-12.0 | 4.5 ± 1.7 / 4 / 3-8.0 | 0.11^a^ |
| Total number of sessions | 13.6 ± 14.7 / 10.0 / 1-72 | 20.2 ± 10.8 / 20.0 / 5-40 | 0.04^a*^ |
| Total duration in minutes | 813.4 ± 2937.6 / 300.0 / 0.5-25200.0 | 792.1 ± 1006.1 / 450.0 / 75.0-3000.0 | 0.14^a^ |

mean ± standard deviation / median / minimum-maximum; ^a^ Wilcoxon rank sum test; ^*^ Significant difference (p≤0.05)

SPEL, studies published in English language; SPNEL, studies published in non-English language
