# Supplementary material for: Publication language and the estimate of treatment effects of physical therapy on balance and postural control after stroke in meta-analyses of randomised controlled trials
Source: PLoS One. 2020 Mar 9;15(3):e0229822. doi: 10.1371/journal.pone.0229822 (PMC7062257; doi:10.1371/journal.pone.0229822)
Supplement: S9 Table — (DOCX) [file pone.0229822.s019.docx]

**S9 Table. Summary of outcome measures**

| Measures of outcomes | SPEL | SPNEL |
| --- | --- | --- |
| Balance |  |  |
| BBS (post-intervention), n (%) | 88 (67%) | 10 (77%) |
| BBS (follow-up), n (%) | 29 (22%) | 2 (15%) |
| PASS (post-intervention), n (%) | 9 (7%) | 2 (15%) |
| PASS (follow-up), n (%) | 6 (5%) | 0 (0%) |
| Autonomy |  |  |
| Barthel index (post-intervention), n (%) | 25 (19%) | 5 (38%) |
| Barthel index (follow-up), n (%) | 11 (8%) | 2 (15%) |
| ADL (post-intervention), n (%) | 2 (2%) | 0 (0%) |
| IADL (post-intervention), n (%) | 0 (0%) | 0 (0%) |
| IADL (follow-up), n (%) | 4 (3%) | 0 (0%) |
| FIM (post-intervention), n (%) | 1 (1%) | 0 (0%) |
| FIM (follow-up), n (%) | 3 (2%) | 0 (0%) |
| Mediolateral and anteroposterior postural deviation (COP, WBA) |  |  |
| WB on paretic limb, EO (post-intervention), n (%) | 22 (17%) | 1 (8%) |
| WB on paretic limb, EO (follow-up), n (%) | 4 (3%) | 0 (0%) |
| WB on paretic limb, EC (post-intervention), n (%) | 2 (2%) | 0 (0%) |
| Mediolateral position of COP, EO (post-intervention), n (%) | 4 (3%) | 2 (15%) |
| Anteroposterior position of COP, EO (post-intervention), n (%) | 3 (2%) | 1 (8%) |
| Anteroposterior position of COP, EO (follow-up), n (%) | 0 (0%) | 1 (8%) |
| Postural stability (COP, LOS) |  |  |
| Sway length EO (post-intervention), n (%) | 12 (9%) | 1 (8%) |
| Sway area EO (post-intervention), n (%) | 10 (8%) | 0 (0%) |
| Sway length EC (post-intervention), n (%) | 9 (7%) | 0 (0%) |
| Velocity COP EO (post-intervention), n (%) | 9 (7%) | 0 (0%) |
| Anteroposterior velocity COP EO (post-intervention), n (%) | 8 (6%) | 0 (0%) |
| Mediolateral velocity COP EO (post-intervention), n (%) | 8 (6%) | 0 (0%) |
| Anteroposterior velocity COP EC (post-intervention), n (%) | 7 (5%) | 0 (0%) |
| LOS, maximum excursion, affected side EO (post-intervention), n (%) | 7 (5%) | 0 (0%) |
| LOS, maximum excursion, anterior, EO (post-intervention), n (%) | 7 (5%) | 0 (0%) |
| LOS, maximum excursion, non-affected side EO (post-intervention), n (%) | 7 (5%) | 0 (0%) |
| Mediolateral velocity COP EC (post-intervention), n (%) | 7 (5%) | 0 (0%) |
| LOS, Movement velocity, affected side EO (post-intervention), n (%) | 6 (5%) | 0 (0%) |
| LOS, Movement velocity, anterior, EO (post-intervention), n (%) | 6 (5%) | 0 (0%) |
| LOS, Movement velocity, non-affected side EO (post-intervention), n (%) | 6 (5%) | 0 (0%) |
| LOS, directional control non-affected side EO (post-intervention), n (%) | 5 (4%) | 0 (0%) |
| LOS, directional control, affected side EO (post-intervention), n (%) | 5 (4%) | 0 (0%) |
| LOS, directional control, anterior, EO (post-intervention), n (%) | 5 (4%) | 0 (0%) |
| LOS, maximum excursion, posterior EO (post-intervention), n (%) | 5 (4%) | 0 (0%) |
| LOS, Movement velocity, posterior EO (post-intervention), n (%) | 5 (4%) | 0 (0%) |
| LOS, directional control posterior EO (post-intervention), n (%) | 4 (3%) | 0 (0%) |
| Sway area EC (post-intervention), n (%) | 4 (3%) | 0 (0%) |
| Velocity COP EC (post-intervention), n (%) | 4 (3%) | 0 (0%) |
| Anteroposterior sway length EO (post-intervention), n (%) | 3 (2%) | 0 (0%) |
| LOS, end-point excursion, affected side, EO (post-intervention), n (%) | 3 (2%) | 0 (0%) |
| LOS, end-point excursion, backward, EO (post-intervention), n (%) | 3 (2%) | 0 (0%) |
| LOS, end-point excursion, forward, EO (post-intervention), n (%) | 3 (2%) | 0 (0%) |
| LOS, end-point excursion, non-affected side, EO (post-intervention), n (%) | 3 (2%) | 0 (0%) |
| Mediolateral sway length EO (post-intervention), n (%) | 3 (2%) | 0 (0%) |
| Anteroposterior variability COP EO (post-intervention), n (%) | 2 (2%) | 1 (8%) |
| Mediolateral variability COP EO (post-intervention), n (%) | 2 (2%) | 1 (8%) |
| Velocity moment EC (post-intervention), n (%) | 3 (2%) | 0 (0%) |
| Velocity moment EO (post-intervention), n (%) | 3 (2%) | 0 (0%) |
| LOS EO (post-intervention), n (%) | 2 (2%) | 0 (0%) |
| LOS, end-point excursion, affected side, EO (follow-up) | 2 (2%) | 0 (0%) |
| LOS, end-point excursion, backward, EO (follow-up) | 2 (2%) | 0 (0%) |
| LOS, end-point excursion, forward, EO (follow-up), n (%) | 2 (2%) | 0 (0%) |
| LOS, end-point excursion, non-affected side, EO (follow-up), n (%) | 2 (2%) | 0 (0%) |
| Maximum COP displacement in anteroposterior direction EO (post- intervention), n (%) | 2 (2%) | 0 (0%) |
| Maximum COP displacement in mediolateral direction EO (post- intervention), n (%) | 2 (2%) | 0 (0%) |
| Stability index EO (post-intervention), n (%) | 1 (1%) | 1 (8%) |
| Velocity COP EC (post-intervention), n (%) | 2 (2%) | 0 (0%) |
| Velocity COP EO (post-intervention), n (%) | 1 (1%) | 1 (8%) |
| Anteroposterior sway length EC (post-intervention), n (%) | 1 (1%) | 0 (0%) |
| COP excursion EO (post-intervention), n (%) | 1 (1%) | 0 (0%) |
| LOS, Ankle strategy, EC (follow up), n (%) | 1 (1%) | 0 (0%) |
| LOS, Ankle strategy, EO (follow up), n (%) | 1 (1%) | 0 (0%) |
| LOS, COG alignment, EC (follow up), n (%) | 1 (1%) | 0 (0%) |
| LOS, COG alignment, EO (follow up), n (%) | 1 (1%) | 0 (0%) |
| LOS, Maximal stability, EC (follow up), n (%) | 1 (1%) | 0 (0%) |
| LOS, Maximal stability, EO (follow up), n (%) | 1 (1%) | 0 (0%) |
| LOS, maximum excursion, affected side EO (follow-up), n (%) | 1 (1%) | 0 (0%) |
| LOS, maximum excursion, anterior, EO (follow-up), n (%) | 1 (1%) | 0 (0%) |
| LOS, maximum excursion, non-affected side EO (follow-up), n (%) | 1 (1%) | 0 (0%) |
| LOS, maximum excursion, posterior EO (follow-up), n (%) | 1 (1%) | 0 (0%) |
| LOS, Movement velocity, affected side EO (follow-up), n (%) | 1 (1%) | 0 (0%) |
| LOS, Movement velocity, anterior, EO (follow-up), n (%) | 1 (1%) | 0 (0%) |
| LOS, Movement velocity, non-affected side EO (follow-up), n (%) | 1 (1%) | 0 (0%) |
| LOS, Movement velocity, posterior EO (follow-up), n (%) | 1 (1%) | 0 (0%) |
| LOS, reaction time, affected side, EO (post-intervention), n (%) | 1 (1%) | 0 (0%) |
| LOS, reaction time, affected side, EO (follow-up), n (%) | 1 (1%) | 0 (0%) |
| LOS, reaction time, backward, EO (post-intervention), n (%) | 1 (1%) | 0 (0%) |
| LOS, reaction time, backward, EO (follow-up), n (%) | 1 (1%) | 0 (0%) |
| LOS, reaction time, forward, EO (post-intervention), n (%) | 1 (1%) | 0 (0%) |
| LOS, reaction time, forward, EO (follow-up), n (%) | 1 (1%) | 0 (0%) |
| LOS, reaction time, non-affected side, EO (post-intervention), n (%) | 1 (1%) | 0 (0%) |
| LOS, reaction time, non-affected side, EO (follow-up), n (%) | 1 (1%) | 0 (0%) |
| Maximum COP displacement in anteroposterior direction EC (post- intervention), n (%) | 1 (1%) | 0 (0%) |
| Maximum COP displacement in anteroposterior direction EC (follow- up), n (%) | 1 (1%) | 0 (0%) |
| Maximum COP displacement in anteroposterior direction EO (follow- up), n (%) | 1 (1%) | 0 (0%) |
| Maximum COP displacement in mediolateral direction EC (post- intervention), n (%) | 1 (1%) | 0 (0%) |
| Maximum COP displacement in mediolateral direction EC (follow-up), n (%) | 1 (1%) | 0 (0%) |
| Maximum COP displacement in mediolateral direction EO (follow-up), n (%) | 1 (1%) | 0 (0%) |
| Mediolateral sway length EC (post-intervention), n (%) | 1 (1%) | 0 (0%) |
| Stability index EC (post-intervention), n (%) | 1 (1%) | 0 (0%) |
| Sway area EC (follow-up), n (%) | 1 (1%) | 0 (0%) |
| Sway length EC (follow-up), n (%) | 1 (1%) | 0 (0%) |
| Sway length EO (follow-up), n (%) | 1 (1%) | 0 (0%) |
| Anteroposterior variability COP EC (post-intervention), n (%) | 0 (0%) | 1 (%) |
| Mediolateral variability COP EC (post-intervention), n (%) | 0 (0%) | 1 (%) |

ADL, activities of daily living; BBS, berg balance scale; COP, center of pressure; Deg, degree; EC, eyes closed; EO, eyes open; FIM, functional independence measure; IADL, instrumental activities of daily living; LOS, limit of stability; PASS, postural assessment scale for stroke; NT, no treatment; s, second; SPEL, studies published in English language; SPNEL, studies published in non-English language; ST, sham treatment; UC, usual care; WB, weight bearing; WBA, weight bearing asymmetry
